# Costs of hospital-acquired *Clostridium difficile* infections: an analysis on the effect of time-dependent exposures using routine and surveillance data

**DOI:** 10.1186/s12962-019-0184-5

**Published:** 2019-08-01

**Authors:** Thomas Heister, Martin Wolkewitz, Philip Hehn, Jan Wolff, Markus Dettenkofer, Hajo Grundmann, Klaus Kaier

**Affiliations:** 1 Institute of Medical Biometry and Statistics, Faculty of Medicine and Medical Center, University of Freiburg, Stefan-Meier-Str. 26, 79104 Freiburg, Germany; 2 Department of Psychiatry and Psychotherapy, Faculty of Medicine and Medical Center, University of Freiburg, Freiburg, Germany; 3 Institute for Hospital Hygiene and Infection Prevention, Gesundheitsverbund Landkreis Konstanz, Radolfzell, Germany; 4 Division of Infection Control and Hospital Epidemiology, University Medical Center Freiburg, Freiburg, Germany

**Keywords:** Hospital-acquired infections, Time-varying exposures, Time-dependent bias, Costs, *Clostridium difficile*

## Abstract

**Background:**

Hospital-acquired infections have not only gained increasing attention clinically, but also methodologically, as a time-varying exposure. While methods to appropriately estimate extra length of stay (LOS) have been established and are increasingly used in the literature, proper estimation of cost figures has lagged behind.

**Methods:**

Analysing the additional costs and reimbursements of *Clostridium difficile*-infections (CDI), we use a within-main-diagnosis-time-to-exposure stratification approach to incorporate time-varying exposures in a regression model, while at the same time accounting for cost clustering within diagnosis groups.

**Results:**

We find that CDI is associated with €9000 of extra costs, €7800 of higher reimbursements, and 6.4 days extra length of stay. Using a conventional method, which suffers from time-dependent bias, we derive estimates more than three times as high (€23,000, €8000, 21 days respectively). We discuss our method in the context of recent methodological advances in the estimation of the costs of hospital-acquired infections.

**Conclusions:**

CDI is associated with sizeable in-hospital costs. Neglecting the methodological particularities of hospital-acquired infections can however substantially bias results. As the data needed for an appropriate analysis are collected routinely in most hospitals, we recommend our approach as a feasible way for estimating the economic impact of time-varying adverse events during hospital stay.

## Background

The incidence of *Clostridium difficile* infections (CDI) has increased dramatically since 2001 [1]. In the United States, CDI was estimated to be responsible for some 453,000 infections and 29,000 deaths in 2011 and, with at least two-thirds of cases considered health care-associated [2], represents a major source of nosocomial infections. In Europe there were some 120,000 cases of healthcare-associated CDI in 2011, with case fatalities ranging from 3 to 30% [3, 4]. CDI has also been found to be associated with large and rising numbers of colitis resulting in colectomies, and increased mortality [5], as well as considerable rates of treatment failure and recurrence [6]. Worries persist about the emergence of more virulent strains of the pathogen [7, 8].

Knowledge of the economic impact of CDI in the hospital setting is of major importance in order to influence behaviour and resource allocation in healthcare facilities, to guide policy makers and to stimulate interest in developing new prevention and treatment strategies [9, 10]. Estimation of the in-hospital costs of CDI, however, is challenging for a number of reasons:

First, hospital-acquired CDI presents a complication occurring in different and often dissimilar groups of patients. As a result, total hospitalization costs of these patients include large amounts of costs that are related to the patients’ main reason for hospitalization.

Second, hospital-acquired CDI often occurs in a relatively late phase of hospitalization, making estimation results ignoring the timing of CDI exposure subject to the time-dependent bias by implicitly assuming that hospital-acquired infections are already present on admission. This bias is always associated with an overestimation of the true effect [11–16].

Correctly accounting for the time-dependency when analysing costs directly is complicated by the general unavailability of daily hospitalization costs, which would allow for an adequate differentiation of pre- and post-infection costs. Third, standard regression methods for continuous endpoints (e.g. costs) do not enable the inclusion of time-dependent covariates.

There is an extensive body of literature on the costs of CDI, which is characterized by the use of heterogeneous settings and statistical methods. A recent systematic review included 45 studies on the costs of both hospital- and community-acquired CDI and found attributable mean costs between $9000 and $30,000 [17]. Another review reported a median cost estimate on hospital-acquired CDI of $9,000, ranging between $3000 and $30,000 and median extra LOS of 7 days ranging from 2.7 to 21.3 days [18].

However, both reviews do not discuss the time-dependency of hospital-acquired CDI. As most included studies are subject to the time-dependent bias, these results are likely an overestimation due to failure to account for the fact that CDI is not present during the entire hospitalization [13, 19]. A recent study in Germany, also ignoring the time-dependency of hospital-acquired CDI, calculated the additional costs, reimbursements and extra LOS of CDI at €6300, €3800 and 10.8 days, respectively [20].

Few studies apply methods to accurately account for the time-varying nature of CDI exposure. Those that do have arrived at more conservative estimates. Stevens et al. using a multistate model, find 2.3 days of extra LOS for a critical care setting. Using a time-to-exposure matching, Tabak et al. find the same effect on LOS of 2.3 days and $6100 attributable costs. To the best of our knowledge there is no study analysing both incremental costs and additional reimbursements of CDI that also takes cost-clustering and the time-dependency of hospital-acquired CDI into account.

The aim of our study is to provide estimates of the impact of CDI on in-hospital costs, reimbursements, and LOS using routinely available data. Special focus is placed on the appropriate consideration of the time-dependent nature of hospital-acquired CDI, the fact that in-hospital costs are highly clustered within diagnostic groups, and the challenge that comorbidities are usually documented as time-fixed. We additionally want to quantify the extent of the time-dependent bias and validate our time-to-exposure stratification approach in a sensitivity analysis.

## Methods

### Setting and data

We use data from the University Medical Center Freiburg (UMCF), a tertiary care teaching hospital in southern Germany. 204,914 complete patient records from 2011 to 2014 are available. These records include age, sex, CDI exposure, main diagnosis, secondary diagnoses, discharge status, LOS, accounting cost and reimbursements.

Accounting costs figures are calculated by the hospital according to standardized methods of the Institute for the Payment system in Hospitals (InEK) system [22]. While intended to provide cost figures as the basis for the national reimbursement calculation of diagnosis-related groups (DRGs), it is also a widely used tool for hospital management purposes due to its highly differentiated patient-based calculation method [23].

Reimbursements are the actual payments the hospital receives for in-hospital treatments. These are based on diagnosis-related groups (DRGs), which is a hospital case classification system for standardized lump-sum reimbursements. These groups are defined by the patients’ diagnoses, gender and age, treatment procedures, comorbidities, and further attributes. Hospitals receive additional reimbursement for every day that a patient stays above the upper length of the stay threshold to compensate for cases requiring unusually long stays. These daily surcharges are however much lower than the mean reimbursement per day below this threshold and designed to not entirely cover additional variable costs to create incentives to reduce length of stay. The German DRG system was implemented in 2003 and applies to all somatic in-patient stays in public and private hospitals.

### Case definition

Over the study period, a total of 559 hospital-acquired cases of CDI were documented by the hospital’s infection control department as part of a German infection surveillance system [24]. Hospital-acquired is defined as having been detected more than 48 h after admission for cases that have no CDI-related main diagnosis.

Absence of CDI-related main diagnosis was defined by excluding the relevant International Classification of Disease (ICD-10) diagnosis, e.g. A04.7: enterocolitis due to *Clostridium difficile*. As main diagnoses are the retrospectively coded principal reason for hospitalization this is to additionally ensure that CDI was in fact hospital-acquired. There are cases which were detected > 48 h after admission with a CDI-related main diagnosis, suggesting that it was already present on admission. These were excluded from the analysis.

For all CDI cases, the time of acquisition of the infection (days since admission) and its duration are available. The date of the diagnostic specimen obtained is used for the time of infection. This dataset is merged with the routine data described above.

### Control selection—background

For the selection of an appropriate control group, we consider three aspects: first, we hypothesize that in-hospital costs (as well as reimbursements and LOS) are highly clustered within diagnostic groups due to the high amount of disease- and procedure-related fixed costs and associated LOS.

Second, the time-dependent nature of hospital-acquired CDI needs to be taken into account to avoid an overestimation of the true effect due to the time-dependent bias [11, 12, 14].

Third, the impact of hospital-acquired CDI on the costs of care may be confounded by comorbidities. Severe cases of nosocomial CDI, however, may also be the cause rather than the consequence of documented comorbidities. This is especially problematic since new comorbidities may be documented during the entire hospital stay, but are recorded only on a time-fixed basis (without information when the secondary diagnosis was acquired or even recorded). Secondary diagnoses are all relevant conditions that are either present on admission but were not the reason for hospitalization or occurred during hospitalization.

We are thus unable to determine whether a documented secondary diagnosis was documented as a comorbidity or as a complication. If a complication occurs as a consequence of CDI, controlling for it may underestimate the true effect, as it should be considered part of the CDI-related burden. Therefore, it is vital to identify comorbidities that cannot occur as a consequence of CDI, but are either cost drivers in their own right, influence the likelihood of CDI, or both [25, 26].

A set of 10 comorbidities (ICD-10, 3 digit secondary diagnoses) was suggested previously with respect to hospital-acquired infections [25, 26]. These 10 comorbidities were identified by an expert panel as being either cost drivers in their own right or to influence the likelihood of an hospital-acquired infection, but impossible to be a consequence of an hospital-acquired infection (see Table 1). These were accordingly used for risk adjustment.

**Table 1:** Descriptive statistics

	1		2		3		4	
					Included in analysis with time-to-exposure restriction			
	All		Patient with CDI relevant main diagnosis		Controls		Cases with nosocomial CDI	
	Mean/N	*SD*	Mean/N	*SD*	Mean/N	*SD*	Mean/N	*SD*
Number of different main diagnosis groups	5878		336		296		296	
Costs (real)	5154	10,895	8278	15,235.38	24,407	36,589	34,749	44,208
Reimbursement (real)	5188	10,479	8198	14,390.32	22,641	34,479	33,515	48,453
Reimbursement-costs (real)	33	3646	− 79	4858.83	− 1765	10,584.12	− 1233	13,145
LOS	7.6	8.86	10.7	11.53	25.7	21.05	34.7	28.1
Days in hospital at the time of CDI acquisition							16.99	16.39
Age	57.70	18.68	62.70	16.46	61.04	16.48	63.61	15.76
CCI	1.90	2.80	3.48	3.32	4.37	3.54	4.83	3.36
Died	2.02%		5.05%		7.43%		11.98%	
Intensive care (h)	104.8	340.8	158.5	439.5	456.8	948.1	806	1352
Comorbidities	N/%		N/%		N/%		N/%	
Number of SD (N)	6.58		9.27		14.91		20.53	
Renal failure (N.18)	7.3%		12.4%		17%		19.6%	
Heart failure (I.50)	2.7%		4.2%		6%		8.2%	
Ischemic heart disease (I.25)	10.1%		14.5%		14%		17.2%	
Diabetes (E.11)	10.9%		15.3%		15%		18.5%	
Hypertension (I.10)	31.7%		37.2%		34%		38.5%	
Atrial fibrillation and flutter (I.48)	7.8%		12.6%		15%		20.5%	
Anaemia (D.62)	5.9%		9.8%		20%		27.5%	
COPD (J.44)	4.1%		6.5%		7%		8.1%	
Cardiac/vascular implants, grafts (Z.95)	8.7%		13.4%		15%		14.7%	
Cancer (C.)	7.4%		10.2%		12%		14.3%	
Observations	204,914		51,857		1951		559	

Column 1 gives the descriptive statistics for all available data. Column 2 restricts it to patients in main diagnosis groups where there is at least 1 case of CDI, all diagnosis groups in which there is no case are excluded. Column 3 shows the controls used in the regression model 2 in Table 2 below. There are 40 main diagnosis groups in which there were no suitable controls for CDI cases when applying the time-to-exposure restriction. Column 4 presents all patients with CDI that are considered in regression model 2 in Table 2. Here, CDI cases which have no suitable controls that have stayed at least as long as the time of CDI exposure are deleted. There are 3 cases of CDI for which no appropriate controls could be found. These cases were therefore excluded from the analyses shown in regression model 2 in Table 2. For 93 out of the 559 cases there were less than 4 controls available for matching. Comorbidities show the 10 comorbidities as defined in Resch (2008). CCI gives the Charlson Comorbidity Score

### Control selection—stratification

**Fig. 1:**
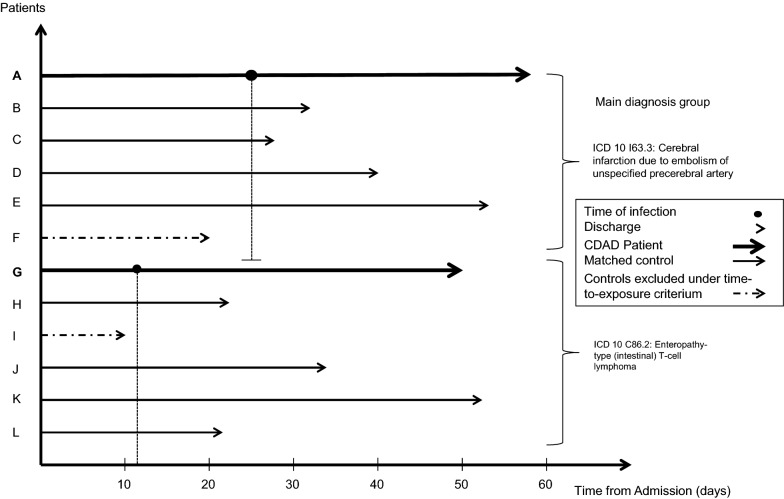
Time to exposure and main-diagnosis matching. This figure illustrates the time-to-exposure stratification and within-main-diagnosis approach used in the regression model. Controls are required to have a length of stay at least equal to the time of infection in days of the corresponding CDI case and be in the same main diagnosis group. Here, B, C, D and E are for instance suitable controls for A, while F–L are not, being in a different main diagnosis group (H–L) or not meeting the time-to-exposure criterion (F). Patients and main diagnosis groups here are chosen exemplary for illustrative purposes

The first two aspects, clustering within diagnostic groups and the time-dependent nature of hospital-acquired CDI cases, are considered using time-to-exposure stratification within the group of patients with the same main diagnosis. Figure 1 illustrates the stratification method. Every patient has one documented main diagnosis per hospitalization episode, representing the retrospectively determined primary reason for hospitalization. We use these main diagnoses (4 digit ICD-10) as a first step for identifying potential unexposed controls.

Some studies have used matching on DRGs to control for cost clustering. However, as DRGs are partly determined by the outcome as well, this introduces a bias to the analysis by conditioning on the future [27]. Time-to-exposure is added as an additional criterion, meaning that the unexposed controls (within the same main diagnosis) are required to have stayed in the hospital at least as long as the exposed CDI cases had stayed before CDI was detected [11]. Out of the eligible controls meeting those criteria, four were randomly chosen for each case. For 93 cases there were less than four controls meeting the matching criteria available.

Our time-to-exposure stratification means that (1) all inpatients unexposed to CDI throughout their hospital stay which could not be matched by their diagnosis code to a CDI-exposed inpatient were excluded, (2) all controls which did not meet the time-to-exposure restriction within the strata were also excluded, (3) only subjects that will never be exposed to CDI are used as unexposed controls, (4) every unexposed control is stratified to a single CDI case only. The analysis therefore includes 559 strata.

**Fig. 2:**
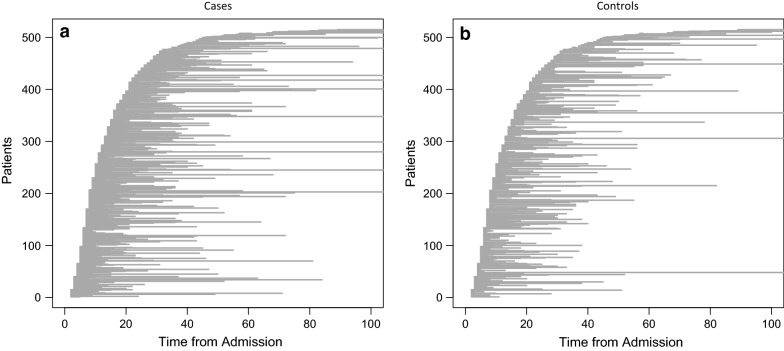
Time of infection and time-to-exposure stratification. This figure shows the time of CDI acquisition, post-exposure length of stay and overall length of stay for patients. Each line represents one patient. **a** Shows all nosocomial CDI cases sorted by infection time in days from admission. The x-axis shows overall length of stay, while the length of a line signifies the post-exposure length of stay. **b** Shows one corresponding control for the cases in **a** which meets the time-to-exposure criterion, that is, they have stayed at least until the infection time of the corresponding case. For illustrative purposes only one of the four matched controls are shown. Figures are truncated at 100 days

Figure 2 illustrates this stratification approach by showing the timing of infection, overall length of stay and post-exposure length of stay of included cases, as well as the length of stay of matched controls in relation to the matching point. It can be seen that only controls that have a length of stay at least equal to the infection time of the cases are included.

### Main analyses

For the main analyses, we chose a generalized linear model (GLM) with a log link and gamma distribution to account for the right-skewed nature of the data [28, 29]. We conduct Modified Park Tests to identify the best fitting distribution, which suggested gamma distributions to be appropriate for costs, reimbursement and length of stay. Our main regression model is therefore

$${\text{lnE}}\left( {y_{i} } \right) = \beta_{0} + \beta_{1} CDAD_{i} + {\mathbf{X}}_{is}\varvec{\beta}_{2s } + \varvec{C}_{ir}\varvec{\beta}_{3r} + \beta_{4} age_{i} + \beta_{5} age_{i}^{2} + \beta_{6} sex_{i}$$

$${\text{with}}\quad Var\left[ {y |x} \right] = \alpha \left[ {E\left( {y |x} \right)} \right]^{2} .$$

Our outcome variable $y_{i}$ is either accounting costs, reimbursements, or LOS. The main variable of interest, $CDAD_{i}$, is a dummy variable indicating hospital-acquired CDI-acquisition for patient *i*. ${\mathbf{X}}_{is}$ is a vector of dummy variables for each above discussed stratum *s* to estimate fixed effects. Comorbidity adjustment is captured by $\varvec{C}_{ir}$, a vector of 10 dummy variables for each relevant comorbidity *r* as suggested by Resch et al. [26] and Noskin et al. [25]. Further baseline risk adjustment is applied by adding sex, age, and age^2^ as covariates.

While the main variable, $CDAD_{i}$, should not be correlated with the unobserved cluster effect as we match four controls to each case, the other explanatory variables likely are. Hausman Tests indicate random effects to be inconsistent. However, the difference in results is small (data not shown), so that for analyses facing smaller sample sizes our approach is likely also feasible with random effects. For all GLM results, both regression coefficients (exponentiated, these represent the CDI-related percentage change in the respective outcomes) and average marginal effects (interpreted as the CDI-related absolute change in the respective endpoint) are shown.

As a sensitivity analysis, the above-described modus operandi for time-to-exposure stratification is evaluated using a cox proportional hazards model and the endpoint LOS. Unlike costs, the endpoint LOS may be analysed using survival models (such as Cox models), which allow the time-dependency of CDI exposure to be taken into account by including CDI exposure as a time-varying covariate. Consequently, we conduct two analyses: First, we analyse the impact of CDI on LOS by including its exposure as a time-varying covariate. Time-to-exposure stratification is not applied, but the cox regression is stratified by main diagnosis. Second, the described time-to-exposure stratification was applied and CDI exposure included as time-fixed covariate of the Cox model. Baseline risk adjustment is applied in both analyses by adding sex, age, age^2^ and the 10 comorbidities discussed above as covariates.

As all patients were either discharged alive or died in the hospital no censoring takes place. Death and discharge alive are considered competing risks when analyzing mortality or risk of acquiring a nosocomial infection. An appropriate consideration of competing risk in the analysis of costs is not possible, and analyzing only those who survived would be conditioning on the future. We therefore included all cases irrespective of death. The resulting two hazard ratios for discharge were compared for consistency and efficiency.

### Pitfalls of multistate modelling

Much of the recent methodological literature discussing the time-dependent nature of hospital-acquired infections has focussed on multistate modelling. In multistate models, the patient’s infection state during hospitalization is modelled by allowing patients to move between different states while hospitalized [30]. These models, however, cannot be employed to directly analyse costs as an endpoint but only LOS, so that to derive an estimate for attributable costs, LOS needs to be multiplied with a daily cost figure.

Additionally, correctly adjusting for confounding is complicated in multistate models. To place our estimates in context with current findings and approaches in the literature, we additionally estimated a simple illness-death type multistate model with three states without comorbidity or main-diagnosis adjustment using 51,857 patients with CDI-relevant main diagnosis (see Appendix: Fig. 4). Extra length of stay was calculated using transition probabilities derived by the Aalen-Johansen estimator [31, 32]. Variance and confidence intervals were calculated using bootstrapping.

Regression analyses are conducted using Stata 14.2 (Stata Corp, College Station, Texas, USA). The multistate model was calculated in R (version 3.4.2, [33] using the etm package.

## Results

### Patient population

Descriptive details regarding the patient selection process are shown in Table 1. Of the 204,914 complete records of patients hospitalized at UMCF between 2011 and 2014 (see column (1) in Table 1), only 51,857 cases are considered for further statistical analyses because of the hypothesized clustering of costs within diagnostic groups (see column (2) in Table 1). Of these 51,857 cases, an additional 49,357 are excluded by only allowing four controls randomly chosen from those that meet the within-diagnosis-time-to-exposure stratification criteria (see column (2), (3) and (4) in Table 1). The need for the time-to-exposure restriction is underlined by the relatively late average time point of CDI exposure (see column (4) in Table 1): the average patient in a CDI relevant main diagnosis group was already discharged (mean LOS 10.7 ± SD days, see column (2) in Table 1) at the average time of CDI exposure (17.0 ± SD days after admission).

### Main regression results

**Fig. 3:**
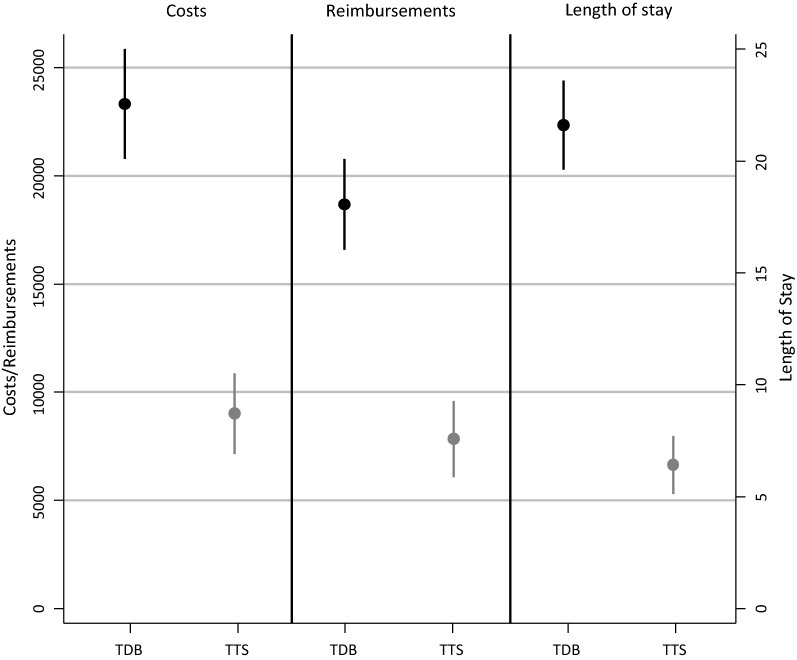
Marginal effects and time-dependent bias. This figure visualizes the marginal effects of the estimation results as given in Table 2. Costs and reimbursements in Euros are measured on the left y-axis, while length of stay is measured in days on the right y-axis. TDB gives the results of the model suffering from the time-dependent bias, corresponding to column 1 in Table 2. TTS gives the results of the correct estimation using the time-to-exposure stratification, corresponding to the results in column 2 in Table 2. The difference between the TDB and TTS estimates equals the size of the time-dependent bias

**Fig. 4:**
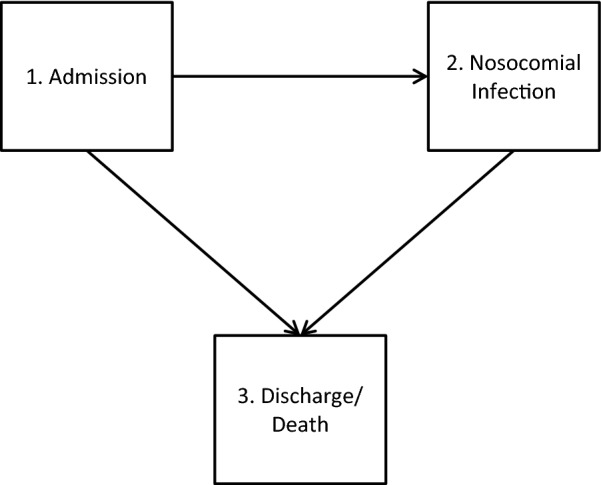
Multistate model. Diagram of multistate model with 3 states. Each patient enters the model at state 0 (hospital admission no CDI infection detected) and may either directly move to state 3 or first to state 2 with defined daily probabilities. At each time-point, each patient occupies 1 of the states. Death and discharge are analysed together, competing risks are not considered

Estimates of the average absolute change in costs, reimbursements, and LOS are shown in Table 2 (rows “marginal effect”) and visualized in Fig. 3. It shows that CDI increases the costs of hospitalization by €9000, leads to €7800 of additional reimbursement, and prolongs the patients’ hospital stay by 6 days (see marginal effects Table 2). All figures are presented in 2014 euros, adjusted using the health care price index of the German Federal Statistical Office [34]. The marginal effects correspond to a 36% (exp(0.31) − 1) increase in the costs of hospitalization, a 33% increase in reimbursements, as well as a 24% prolongation of hospital stay.

**Table 2:** Main regression results

Outcome variables	(1)	(2)
	Ignoring time-dependent CDI exposure	Using time-to-exposure stratification
Costs	1.08*** [1.00;1.15]	0.31*** [0.25;0.37]
Marginal effect	23,313*** [20,772;25,854]	9016*** [7152;10,880]
Reimbursement	0.94*** [0.87;1.01]	0.29*** [0.23;0.352
Marginal effect	18,678*** [16,580;20,776]	7838*** [6072;9605]
Extra length of stay	0.96*** [0.87;1.02]	0.22*** [0.18;0.26]
Marginal effect	21.6*** [19.6;23.6]	6.4*** [5.1;7.8]
*N*	2709	2500

This table summarizes a total of six GLM regression analyses, each using a gamma distribution and a log link function. Each cell gives the effect of CDI infection on the respective outcome on the left from different GLM regressions. All regressions use a within-main-diagnosis estimation and control for comorbidities according to Resch (2008), for age, age^2^, and for sex. Regressions in the second column additionally apply a time-to-exposure stratification. 95% confidence intervals are in brackets

* *p* < 0.1, ** *p* < 0.05, *** *p* < 0.01

As shown in Table 2, the impact of time-to-exposure stratification on the estimated effect of CDI is substantial. Ignoring exposure time leads to a more than twofold overestimation. The estimates for the additional hospitalization costs are then €23,000, €18,000 for reimbursements, and 21 days for CDI-related prolonged LOS. Relative effects are similarly much larger, being 190% (exp(1.08) − 1), 156%, and 160% for costs, reimbursements, and LOS respectively (see column (1) in Table 2). The difference between these estimates (column (1) and (2) in Table 2) equals the size of the time-dependent bias [11, 12, 14].

### Cox regression models

Results of the two Cox regression analyses are presented in Table 3. Hazard ratios for time to discharge (alive or due to death) were calculated. We compare model 1, stratified by main diagnosis where CDI exposure is included as a time-varying covariate with model 2, in which we apply time-to-exposure stratification and included CDI exposure as a time-fixed covariate. Both models show a hazard ratio significantly lower than one, meaning that CDI exposure is associated with lower hazard of being discharged, implying an increased LOS [35]. In contrast to the results above, both specifications appropriately address the time-dependent nature of hospital-acquired CDI. The hazard ratios of the two specifications are similar yet not identical (HR 0.74 and HR 0.67), but the variance of these two estimates is almost identical (standard error 0.035 and 0.037, respectively), indicating no substantial loss in efficiency due to the unavoidable decrease in the number of cases after time-to-exposure stratification (see Table 3).

**Table 3:** Sensitivity analysis: Cox regressions

	(1)	(2)
	Time-varying CDI exposure, no time-to-exposure stratification	Time-fixed CDI exposure, time-to-exposure stratification
Hazard ratio	0.669*** (0.0366)	0.741*** (0.0348)
N	52,679	23,052

Each cell gives the effect of CDI infection on the time to discharge (alive or due to death) from different Cox proportional hazard regressions. A hazard ratio < 1 indicates an increased length of stay due to CDI exposure. Due to convergence issues in the regression model, the amount of controls per case is not restricted to four in this case. All regressions use a within-main-diagnosis estimation by stratifying the regression and control for comorbidities according to Resch (2008), for age, age^2^, and for sex. Standard errors in parentheses

* p < 0.1, ** p < 0.05, *** p < 0.01

### Multistate models

Using the multistate model we found an extra LOS of 5.36 (95% CI 3.9–6.81). To be able to analyse the endpoint costs, some studies use average daily costs and multiply them with extra LOS estimates derived from a multi-state model [36–38]. For Germany, a possible constant daily cost would be €575.9 [39], which multiplied by the estimated 6.4 days of additional LOS from our time to exposure analysis would lead to €3680 of CDI-related additional costs.

## Discussion

The results of our study may be interpreted from different perspectives: from a clinical perspective, CDI exposure exacerbates illness, prolonging hospital stay by 6 days. From the broader healthcare perspective, this is accompanied by additional costs, which amount to about €9000 per CDI case. From the third party payer’s perspective, CDI cases lead to additional expenses in the form of reimbursement of about €7800. Additional reimbursement for CDI compared to controls can be explained by the current structure of the DRG system. Using our within-main diagnosis stratification process one might expect that reimbursement should not be higher for cases than controls in a diagnosis-driven reimbursement system. However, while principally a lump-sum reimbursement system based on the diagnosis and procedure, the DRG system also contains LOS-related elements. Hospitals receive additional reimbursement per day for patients staying longer than the upper LOS threshold defined per DRG. The additional daily surcharges are below incremental daily costs to create an incentive for reducing length of stay [23]. Furthermore, additional reimbursement is possible for very complex intensive care treatments [40].

From the perspective of the hospital administration, the impact of CDI exposure may be summarized by the difference between additional costs and reimbursements. On average, €1200 of the additional costs of CDI are not covered by additional reimbursements from insurance companies. Our estimates suggest that hospitals have a financial incentive to reduce nosocomial CDI cases. Using accounting costs presents the long run perspective, as fixed costs are allocated to cases based on surrogate measures of resource utilization. However, as the fixed costs cannot be recouped in the short run if CDI is avoided, to reflect decision making from the hospital management perspective it may be more appropriate to only consider the variable costs [41].

Our estimates are somewhat higher than those of previous studies that employed time-dependent methods but lower than those of previous studies that ignored time-dependency [15, 17, 18, 21]. However, these studies mostly focused on the U.S., making comparison problematical. Compared to a recent study for Germany, we found similar, slightly higher estimates despite that study ignoring the time-dependency [20]. The reason is unclear, as our estimates using a similar method that ignores the time-dependency are substantially larger. It is possible that by matching on DRG they underestimate the effect by conditioning on the future, as DRGs are partly determined by outcomes and cannot be used to reflect baseline risk [42]. This may outweigh the overestimation caused by the time-dependent bias.

In comparison to our main estimates, using an approach with LOS obtained from a multistate model multiplied with daily costs leads to a substantial underestimation of the effect on costs. Presumably this is because multiplying extra LOS with average daily costs neglects the increased care intensity after CDI exposure. As this method is increasingly used to calculate the additional costs of HAIs, this underestimation warrants further analysis [36–38]. Choosing a daily costs figure that reflects actual resource utilization after CDI exposure is imperative. Interestingly, despite failing to account for comorbidities and main diagnosis clustering, the 5.3 days of extra LOS derived from the multi-state model is close to the 6.4 days from our fully adjusted model, indicating that at least in terms of LOS the time-dependency of the exposure may play the largest role in biasing results. Future research should aim to disentangle and quantify the different biases in more detail.

By showing the substantial effect of the time-dependent bias we are in line with recent methodological studies [11, 12, 14, 37]. Even the magnitude of the time-dependent bias is similar to previous results [43].

Our study has several limitations. First, it is based on administrative data, and diagnosis coding errors are inevitable. With respect to the analysed infections it is moreover important to stress that we only investigate CDI cases that were both hospital-acquired and detected during the same period of hospitalisation. This only represents a part of all CDI cases, as there are not only many community-acquired cases but also hospital-acquired cases that were not detected during the same stay but resulted in readmissions for CDI. While the latter are also hospital-acquired cases, we were unable to distinguish them from community-acquired ones and did therefore not analyse them. Our sample might therefore be biased towards more expensive cases with longer LOS, as the possibility of detecting a CDI during a hospital stay might be a correlated with length of stay. Cases with CDI present on admission (N = 112) had average total costs of hospitalization of €5700 with a LOS of 12 days (data not shown) compared to €35,000 and 34 days for nosocomial cases (see Table 1).

We analysed patients hospitalized at a single centre, so that the generalizability of our findings may be limited. However, the reimbursement system is the same across Germany, and the cost calculation is a standardized method used by 340 German hospitals [22]. Moreover, many hospitals participate in a program using a standardized pathogen surveillance system [24]. The proposed methods are therefore likely applicable in most German hospitals. Nonetheless, as infection prevention and control methods, CDI incidence and costs structures differ between hospitals in Germany, results may be different for other hospitals [44].

Technically, the applied time-to exposure stratification process includes conditioning on the future: only subjects that will never be exposed to CDI are used as unexposed controls [11]. Due to the high number of potential controls and the rarity of the exposure, however, this detail should be of minor relevance.

By limiting the number of controls per case to four—following a general rule of case–control studies—we are potentially losing useful information. However, allowing the number of unexposed controls stratified to a single CDI case to vary may cause issues because early CDI cases (e.g. detected at day 5 after hospital admission) are associated with many more controls than CDI cases that occurred later (e.g. detected at day 30 after hospital admission, a time point at which most potential controls were already discharged and therefore excluded from the analysis). The potentially systematic decrease in the number of controls with increasing time points of CDI onset could then influence the results. Future research should address this issue by developing methods for the identification of the time of CDI acquisition as an effect modifier, an easy way for balancing the number of controls per CDI case and/or the identification of a maximum number of controls necessary to efficiently estimate the main effect.

## Conclusions

While there is an increasing body of literature taking into account the methodological challenges of HAI, most fail to consider all of the discussed issues. Combining existing methods, our approach provides a useful way to account for time-varying exposures, baseline confounding, and cost-clustering at the same time. As the data needed for this analysis are collected routinely in most hospitals we believe that the proposed approach is a feasible way of analysing the economic impact of time-varying adverse events during hospital stay. This does not only apply to hospital-acquired infections but also to other in-hospital adverse events whose probability of occurrence or detection is a function of the LOS.

## Data Availability

The datasets analyzed during the current study are not publicly available due to German data protection regulations but are available from the corresponding author on reasonable request.

## Appendix

See Fig. 4.

## Abbreviations

CDI: *Clostridium difficile* infection
DRG: diagnosis-related groups
GLM: generalized linear model
HAI: hospital-acquired infections
LOS: length of stay
UMCF: University Medical Center Freiburg
